# Public acceptance and uptake of oesophageal adenocarcinoma screening strategies: A mixed-methods systematic review

**DOI:** 10.1016/j.eclinm.2022.101367

**Published:** 2022-04-04

**Authors:** Jasmijn Sijben, Yonne Peters, Kim van der Velden, Linda Rainey, Peter D. Siersema, Mireille J.M. Broeders

**Affiliations:** a Department of Gastroenterology and Hepatology, Radboud Institute for Health Sciences, Radboud university medical center, Geert Grooteplein-Zuid 8, Nijmegen 6500 HB, the Netherland; b Radboud Institute for Health Sciences, Radboud University Medical Center, Geert Grooteplein-Zuid 8, Nijmegen 6500 HB, the Netherland; c Dutch Expert Centre for Screening, Wijchenseweg 101, Nijmegen 6538 SW, the Netherland

**Keywords:** Esophageal Neoplasms, Barrett Esophagus, Mass screening, Early detection of cancer, Endoscopy, Diagnostic Techniques, Digestive System, Patient Acceptance of Health Care, Patient reported outcome measures, Patient preference, Patient participation, Qualitative Research

## Abstract

Oesophageal adenocarcinoma (OAC) is increasingly diagnosed and often fatal, thus representing a growing global health concern. Screening for its precursor, Barrett's oesophagus (BO), combined with endoscopic surveillance and treatment of dysplasia might prevent OAC. This review aimed to systematically explore the public's acceptance and uptake of novel screening strategies for OAC. We systematically searched three electronic databases (Ovid Medline/PubMed, Ovid EMBASE and PsycINFO) from date of inception to July 2, 2021 and hand-searched references to identify original studies published in English on acceptability and uptake of OAC screening. Two reviewers independently reviewed and appraised retrieved records and two reviewers extracted data (verified by one other reviewer). Of the 3674 unique records, 19 studies with 15 249 participants were included in the review. Thematic analysis of findings showed that acceptability of OAC screening is related to disease awareness, fear, belief in benefit, practicalities and physical discomfort. The findings were mapped on the Integrated Screening Action Model. Minimally invasive screening tests are generally well-tolerated: patient-reported outcomes were reported for sedated upper endoscopy (tolerability ++), transnasal endoscopy (tolerability +), tethered capsule endomicroscopy (tolerability +/-), and the Cytosponge-TFF3 test (acceptability ++). In discrete choice experiments, individuals mainly valued screening test accuracy. OAC screening has been performed in trials using conventional upper endoscopy (*n* = 231 individuals), transnasal endoscopy (*n* = 966), capsule endoscopy (*n* = 657) and the Cytosponge-TFF3 test (*n* = 9679), with uptake ranging from 14·5% to 48·1%. Intended participation in OAC screening in questionnaire-based studies ranged from 62·8% to 71·4%. We conclude that the general public seems to have interest in OAC screening. The findings will provide input for the design of a screening strategy that incorporates the public's values and preferences to improve informed participation. Identification of a screening strategy effective in reducing OAC mortality and morbidity remains a crucial prerequisite.

**Funding:**

This study was funded by the Netherlands Organization for Health Research and Development (ZonMw) under grant 555,004,206.

## Introduction

Oesophageal cancer is the 6th most common cause of cancer death worldwide.^1^ The main subtypes, oesophageal adenocarcinoma (OAC) and oesophageal squamous cell carcinoma (OSCC), have different epidemiological features.^2^ The incidence of OAC has surpassed the incidence of OSCC in the US, Canada, Australia and northwest Europe.^2^, ^3^, ^4^ Symptoms such as dysphagia only manifest when the tumour has grown substantially. Hence, OAC has often metastasised to lymph nodes and distant organs before symptomatic presentation, resulting in a dismal prognosis (overall 5-year survival is less than 20%).^5^ High-risk countries have therefore started a discussion on whether OAC could be prevented or detected earlier through screening. Besides identifying a beneficial screening strategy, concurrent assessment of the public's willingness to undergo screening is essential.^6^

OAC is thought to develop mainly in the precursor lesion Barrett's oesophagus (BO).^5^ OAC can be prevented by endoscopic treatment of low-grade or high-grade dysplasia in BO.^7^ Endoscopic surveillance of BO is recommended by societal guidelines to detect these treatable lesions in a timely manner.^8^, ^9^, ^10^ However, the problem is that >90% of OAC cases develop in individuals without a previously known BO diagnosis.^11^ Screening endoscopy-naive individuals for the presence of BO and related neoplasia, coupled with surveillance and treatment interventions if BO or dysplasia is detected, might help to prevent or early detect and endoscopically treat OAC.

As the population benefit of potential OAC screening mainly depends on the public's participation, insights into factors that drive individuals to take up the invitation or to decline it are needed. Introducing a novel screening strategy will expose the public to several potential harms, e.g., side-effects of the test, psychological consequences, and overdiagnosis. Exploration of individuals’ perceptions of OAC screening and how they weigh potential benefits and harms is therefore crucial.

We systematically reviewed the literature on acceptability of OAC screening and available screening tests from the perspective of the target population (broadly defined as individuals with or without gastro-oesophageal reflux disease [GORD] or other risk factors for BO and OAC, due to the current lack of consensus on selection methods). Our second aim was to systematically summarize studies reporting uptake of OAC screening in trials or intended participation in screening.

## Methods

This systematic literature review was performed and reported according to the recommendations of the Preferred Reporting Items for Systematic Reviews and Meta-Analyses (PRISMA) criteria.^12^ The protocol for this review has been registered in PROSPERO (CRD42021239232).

### Search strategy and selection criteria

Three databases, Ovid Medline/PubMed, Ovid EMBASE and PsycInfo, were searched from their date of establishment to July 2, 2021. Keywords used in the search included a combination of English and American spellings of Barrett's oesophagus, oesophageal neoplasm or adenocarcinoma, mass screening, early detection of cancer, endoscopy, cytosponge, breath analysis, attitude to health, decision making, patient acceptance of health care, patient-reported outcomes, patient preference, public opinion, patient participation, uptake and qualitative research. The search strategies (appendix pp 3–8) were developed in consultation with an experienced medical information specialist. To identify any additional relevant studies, we hand-searched reference and citation lists of included studies and relevant review papers.

Quantitative, qualitative, or mixed design studies were included in the review (detailed description of eligibility criteria in appendix p 2). Studies were required to have a sample of individuals with or without GORD and/or other risk factors for BO or OAC, who were invited to undergo OAC screening or who were hypothetically offered OAC screening. Studies were required to report on screening acceptability and/or uptake. Studies were excluded from the review if they were not published in English or Dutch, were not peer-reviewed, were (conference) abstracts, were not original research studies, only included patients with a previous diagnosis of OAC or associated (pre)cancerous lesions, or evaluated screening for other oesophageal conditions. Study authors were contacted if the full text manuscript was unavailable in our institution. The selection process was piloted by applying the selection criteria to a sample of papers (*n* = 200). After that, two researchers independently screened each title, abstract and full text for eligibility (JS and KvdV).

### Outcomes and definitions

Our definition of OAC screening was deliberately broadened to evaluate the full scope of the concept; offering a screening exam (i.e., sedated or unsedated upper endoscopy, ultrathin transnasal endoscopy, video capsule endoscopy, non-endoscopic cell collection device, blood test, or breath analysis) in a community, primary, secondary, or tertiary care setting, aimed at detection of BO or early stage OAC, followed by surveillance of BO and treatment of BO-related neoplasia. We refer to this definition throughout our manuscript as “OAC screening”, thus including screening for BO. The primary outcome of interest was the acceptability of OAC screening, which was predefined to include perceived threats (perceived susceptibility vs perceived seriousness), perceived benefits and harms of screening and how these are weighed by the target screening population, willingness to undergo the procedure again afterwards, preferred screening test, and tolerability of screening tests. Additional outcomes of interest were uptake of OAC screening (in screening trials) or intended participation (in survey studies).

### Data collection and analysis

Two reviewers (JS and KvdV) independently extracted data from each study, one other reviewer (YP) verified the data. We extracted data on study setting, recruitment methods, applied screening tests, study subjects’ characteristics (including age, gender, ethnicity, GORD symptoms, presence of familial BO/OAC, civil status, employment status, education, risk behaviour), acceptability outcomes, and the number of study subjects participating or intending to participate in OAC screening.

We used the EPPI‐Centre tool to assess the quality of qualitative studies.^13^ The methods for sampling, data collection and analysis were categorised as: high quality (thorough attempts were made to increase rigour), medium quality (some steps were taken), or low quality (minimal steps). Studies were also scored on the extent to which the findings were grounded/supported by the data, contributed either depth or breadth of findings (in relation to their ability to answer the review question) and privileged the perspectives and experiences of people.

Assessing the quality of discrete choice experiments (DCEs) in healthcare is a relatively recent methodological area. We used the ISPOR checklist,^14^ which consists of ten items: defining the research question, attributes and levels, construction of tasks, experimental design, preference elicitation, instrument design, data collection, statistical analyses, results and conclusions, and study presentation. Each item has a main question and three sub questions, which may also have more than one component. The ISPOR checklist is not designed to produce a quality score based on study characteristics, but instead is intended as a means to highlight methodological aspects and their reporting.

The STROBE initiative checklist^15^ was adapted to fit studies examining patient-reported outcomes and assessed quality of reporting, sample size, measurement instruments, statistical analysis on eight domains. Each domain was coded as 1 (yes) or 0 (no) with total risk-of-bias scores ranging from 0 to 8. Scores <6, 6–7, and >7 were considered as low, fair, and good, respectively.

Each of the identified studies was appraised by two independent reviewers (JS, KvdV). Any disagreements on inclusion, data extraction or quality assessment were resolved by discussion or consulting a third reviewer (YP, LR, PS, MB).

Qualitative data were analysed using Braun and Clarke's approach, i.e., familiarisation with the data, organising data into meaningful groups using a data-driven strategy, developing factors by evaluating overarching topics and relationships, and studying the interconnectedness of the topics.^16^ This led to the identification of qualitative factors, which were subsequently linked to the Integrated Screening Action Model (I-SAM).^17^ This model was developed to support understanding of screening behaviour and to identify targets for intervention. There are three key aspects to the I-SAM: (1) a sequence of stages that people pass through in engaging in screening behaviour, (2) screening behaviour is shaped by the interaction between participant and environmental influences, and (3) targets for intervention should focus on the sources of behaviour (capability, opportunity, and motivation).

We intended to present pooled proportions of screening trial uptake, stratified by screening test. However, performing a meta-analysis was not possible due to high heterogeneity in the recruitment methods (telephone vs letter, using reminders), timing of eligibility screening in studies (excluding participants before vs after they agreed to participate), and invited population. Meta-analysis of patient-reported outcomes was also not possible due to inconsistency in range, direction and wording of questionnaires, measurement timing, and sedation. Quantitative findings were therefore presented in tables (Table 3 and appendix pp 16–20), summarized narratively, and included in Figure 2.

### Role of the funding source

The funder of the study had no role in study design, data collection, data analysis, data interpretation, or writing of the report. All authors had full access to all the data in the study and had final responsibility for the decision to submit for publication.

## Results

**Figure 1:**
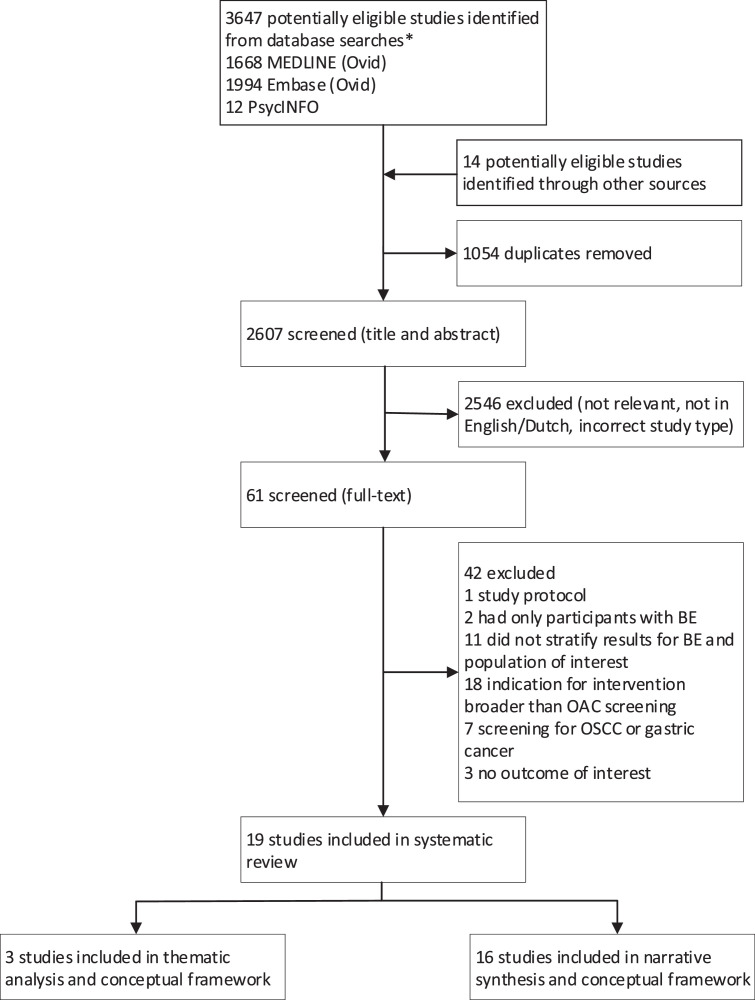
Flow chart summarizing study identification and selection.

Our database searches identified 3674 potentially eligible studies. Study selection and reasons for exclusion are summarised in Figure 1 (reasons for exclusion per study are in appendix pp 9–11). Overall, 19 studies were included (Table 1).

**Table 1:** Study characteristics.

Author (year), country	Study type	Setting	Population	Sample size, n	Screening test	Scenario	Acceptability /uptake primary study focus	Outcome(s) of interest	Study quality
Qualitative									
Freeman et al. (2017), UK^28^	Semistructured interviews, focus groups	Community sample	50–69 y, GORD symptoms/PPI records*	33	Cytosponge-TFF3	Hypothetical	Yes	Acceptability	Fair
McGoran et al. (2019), UK^30^	Semistructured interviews	Secondary care	Referral for dyspepsia	4	TNE, EGD	Experienced	Yes	Expectations and experiences	Good
Tan et al. (2019), UK^31^	Cross-sectional analysis	Facebook community	NR	NR	Cytosponge-TFF3	Hypothetical	Yes	Public perspective and barriers towards uptake	Medium

Mixed methods									
Peters et al. A (2020), The Netherlands^34^	DCE	Population registry sample	50–75 y	375	2 unlabelled hypothetical tests	Hypothetical	Yes	Screening preferences, intended participation	No methodological deficiencies
Peters et al. B (2020), The Netherlands^33^	DCE	Population registry sample	50–75 y	554	EGD, TNE, non-endoscopic device, breath/ blood test	Hypothetical	Yes	Screening preferences, intended participation	No methodological deficiencies

Quantitative									
Blevins et al. (2018) US^18^	RCT	Random sample Olmsted County residents	>50 y, with GORD symptoms	201	huTNE, muTNE, EGD	Experienced	Yes	Screening preferences, tolerability	Fair
Chak et al. (2014), US^19^	RCT	Veterans primary care network	45 - 85 y, veterans, with or without GORD	184 (1210 invited)	TNE, ECE	Experienced	Yes	Uptake, tolerability	Fair
Chang et al. (2011) US^20^	Randomized pilot study	Random sample Olmsted County residents	>50 y, with GORD symptoms	60 (185 invited)	TNE, ECE, EGD	Experienced	Yes	Uptake, tolerability, anxiety	Fair
Eliakim et al. (2004) Israel^35^	Cohort	Secondary care	GORD patients	17	ECE, EGD	Experienced	No (detection rate)	Modality preference, tolerability	Low
Essink et al. (2007), The Netherlands^32^	Case-control	Secondary/tertiary care	Referral for upper GI symptoms	214	EGD	Experienced	Yes	Tolerability, anxiety	Fair
Fitzgerald et al. (2020), UK^27^	RCT	109 general practices UK	>50 y, PPI records*	1654 (6983 invited)	Cytosponge-TFF3	Experienced	No (detection rate)	Uptake, acceptability	Good
Gora et al. (2016), US^21^	Cohort	1 primary care practice	>18 y, with or without GORD/ other risk factors	20	TCE	Experienced	No (feasibility)	Tolerability	Low
Gupta et al. (2014), US^22^	Cross-sectional survey	Community sample MN	> 50 y, with GORD symptoms	136	TNE, ECE, EGD	Hypothetical	Yes	Knowledge, attitudes, preferences and intended participation	Fair
Kadri et al. (2010), UK^29^	Cohort	12 general practices UK	50–70 y, PPI records*	504 (2696 invited)	Cytosponge-TFF3	Experienced	No (sensitivity, specificity)	Uptake, acceptability, test-induced distress, anxiety	Good
Mori et al. (2011), Japan^36^	Cohort	Secondary/tertiary care	Upper GI symptoms	1580	TNE, UUE, EGD	Experienced	No (diagnostic capability)	Tolerability	Fair
Peery et al. (2012), US^23^	Cohort	Primary care network	40–85 y	426	TNE	Experienced	No (procedure yield)	Tolerability	Fair
Ramirez et al. (2008), US^26^	Cohort	Veterans Affairs Medical centre	Veterans with GORD	100	ECE, EGD	Experienced	No (diagnostic yield)	Tolerability, modality preference	Low
Sami et al. (2015), US^24^	RCT	Random sample Olmsted County residents	>50 y, with GORD symptoms	209 (459 invited)	huTNE, muTNE, EGD	Experienced	Yes	Uptake	Fair
Wilkins et al. (2005), US^25^	Cohort	1 primary care practice	> 18 years, persistent GORD symptoms	56	UUE	Experienced	No (feasibility)	Willingness to undergo the procedure	Fair

*NR*, not reported; *OAC*, oesophageal adenocarcinoma; *RCT*, randomized controlled trial; *DCE*, discrete choice experiment; *GORD*, gastro-oesophageal reflux disease; *PPI*, proton-pump inhibitor; *huTNE*, in clinic unsedated transnasal endoscopy; *muTNE*, mobile-based unsedated transnasal endoscopy; *EGD*, esophagogastroduodenoscopy; *TNE*, transnasal endoscopy; *ECE*, oesophageal capsule endoscopy; *TCE*, tethered capsule endomicroscopy; *UUE*, unsedated ultrathin endoscopy.

* Or other prescribed acid-suppressant therapy.

### Characteristics of included studies

The included studies were published between 2004 and 2020 and were performed in the United States (US) (*n* = 9),^18^, ^19^, ^20^, ^21^, ^22^, ^23^, ^24^, ^25^, ^26^ United Kingdom (UK) (*n* = 5),^27^, ^28^, ^29^, ^30^, ^31^ the Netherlands (*n* = 3),^32^, ^33^, ^34^ Israel (*n* = 1),^35^ and Japan (*n* = 1).^36^ Study participants experienced a real-life screening procedure (*n* = 14),^18^, ^19^, ^20^, ^21^^,^^23^, ^24^, ^25^, ^26^, ^27^^,^^29^^,^^30^^,^^32^^,^^35^^,^^36^ or completed a questionnaire or interview on their intent to participate in OAC screening (*n* = 5).^22^^,^^28^^,^^31^^,^^33^^,^^34^ Study designs included cohort studies (*n* = 7),^21^^,^^23^^,^^25^^,^^26^^,^^29^^,^^35^^,^^36^ a case-control study (*n* = 1),^32^ randomised controlled trials (RCTs) (*n* = 5),^18^, ^19^, ^20^^,^^24^^,^^27^ qualitative studies (*n* = 3),^28^^,^^30^^,^^31^ DCEs (*n* = 2),^33^^,^^34^ and a survey (*n* = 1).^22^ The DCE design is a form of trade-off analysis that is increasingly used in healthcare research. One included DCE was unlabelled^34^ (i.e., using generic screening test characteristics), while the other DCE was labelled (i.e., mentioning the actual screening test in each choice option).^33^

Studies investigated a variety of OAC screening tests. Ten studies assessed conventional upper endoscopy, which is currently the gold standard for both screening and surveillance of BO.^18^^,^^20^^,^^22^^,^^24^^,^^26^^,^^30^^,^^32^^,^^33^^,^^35^^,^^36^ Several studies investigated less-invasive endoscopic alternatives: ultrathin transnasal endoscopy (*n* = 9),^18^, ^19^, ^20^^,^^22^, ^23^, ^24^^,^^30^^,^^33^^,^^36^ ultrathin oral endoscopy (*n* = 2),^25^^,^^36^ and oesophageal capsule endoscopy, which avoids insertion of an endoscope (*n* = 5).^19^^,^^20^^,^^22^^,^^26^^,^^35^ One study assessed tethered capsule microendoscopy, in this case the oesophageal capsule implements optical coherence tomography to collect microscopic images of the oesophagus.^21^ The Cytosponge-TFF3 is a non-endoscopic ingestible oesophageal sampling device and was investigated in four studies.^27^, ^28^, ^29^^,^^31^ One study addressed the use of a blood or breath test.^33^ One DCE used hypothetical screening test scenarios.^34^

### Quality of the evidence

A detailed risk of bias summary is reported in the appendix (pp 12–15). Threats to rigour in qualitative studies were small^28^^,^^30^ to moderate in one study (no steps taken to increase rigour in sampling and data collection).^31^ DCE studies had no to minor methodological deficiencies.^33^^,^^34^ Studies measuring patient-reported outcomes had medium to low risk of bias; main shortcomings were a lack of information on non-responders and using unvalidated instruments.^18^, ^19^, ^20^, ^21^^,^^23^, ^24^, ^25^^,^^27^^,^^29^^,^^32^^,^^35^^,^^36^ Three included studies were authored by co-authors on this paper.^32^, ^33^, ^34^ These papers were therefore assessed by other reviewers (JS and KvdV).

### Acceptability of OAC screening

**Figure 2:**
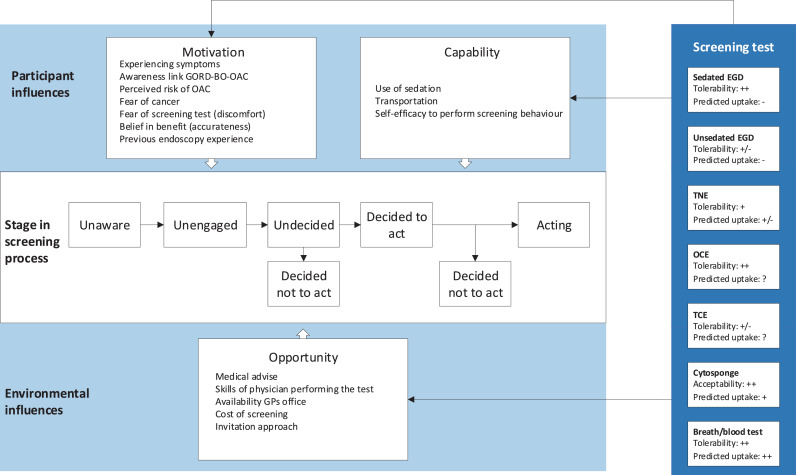
Integrated Screening Action Model focussed on OAC.^17^ Predicted uptake was based on one included discrete choice experiment.^33^ Physical discomfort scores were based on patient-reported outcomes in included studies; -/-, very low tolerability; –, low tolerability; +/-, medium tolerability; +, high tolerability; ++, very high tolerability (appendix pp 20). *GORD, gastro-oesophageal reflux disease; BO, Barrett's oesophagus; OAC, oesophageal adenocarcinoma; EGD, esophagogastroduodenoscopy; TNE, transnasal endoscopy; OCE, oesophageal capsule endoscopy; TCE, tethered capsule endomicroscopy.*

**Figure 3:**
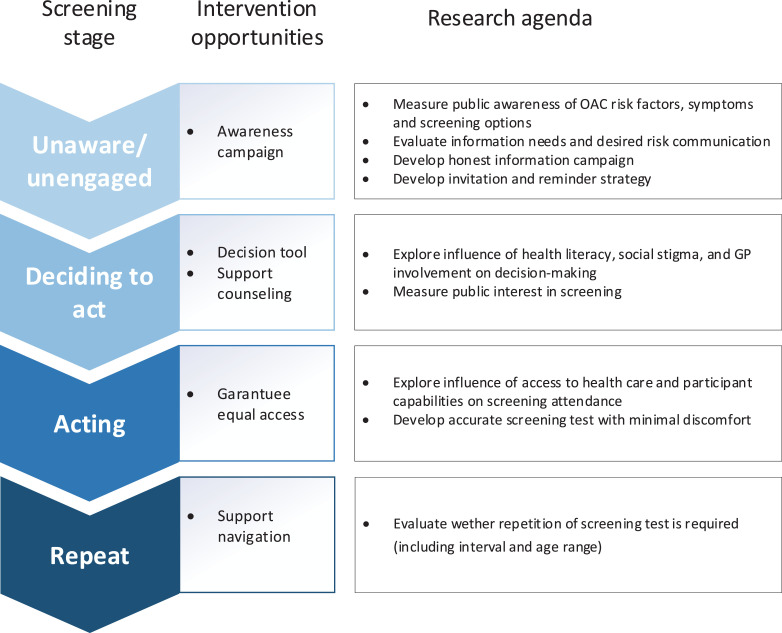
Research directions for further study of the public's perspective on OAC screening, identified through comparison of review findings with the I-SAM.^17^

Table 2 shows the key factors associated with acceptability of OAC screening derived from qualitative studies and how these relate to the I-SAM. Quantitative measures of acceptability were: tolerability/pain/gagging/choking/anxiety/acceptability scores,^18^, ^19^, ^20^, ^21^^,^^23^^,^^26^^,^^27^^,^^29^^,^^32^^,^^36^ willingness to undergo the procedure again,^18^^,^^20^ preferred screening test,^22^^,^^26^^,^^35^^,^^36^ and trade-offs in decision making^18^^,^^33^^,^^34^ (details are in the appendix pp 16–20). An integrative summary of both qualitative and quantitative findings per stage in the screening process is provided below and in Figure 2, relating these findings to key constructs in the I-SAM (in italics) Figure 3. shows research gaps that were identified after comparison of the data with the I-SAM.

**Table 2:** Thematic analysis of factors associated with acceptability of OAC screening and their relation to key constructs in the I-SAM.

Screening stage	Factors	Context	Exemplary quote	I-SAM constructs
Awareness	Association GORD, BO, OAC	All	*"I've never taken it [GORD] to the next step in my mind" ^28^*	Perceived risk (motivation), Knowledge (capability)
Engagement	Risk and consequence of OAC	All	*"My father died from Oesophageal cancer in 2015. As it doesn't really show any symptoms until it is too late to treat it he was gone less than 8 weeks after diagnosis." ^31^*	Perceived risk/emotions (motivation)
Decision to act	Fear of cancer diagnosis	All	*"If you're reading something with ‘cancer’, you're frightening them anyway… You say cancer, people won't take the pill" ^28^*	Emotions/perceived risk (motivation)
	Fear of complications	Cytosponge-TFF3	*“What if it got stuck? Because you know sometimes when a sweet goes down the wrong way…and it gets stuck? That is scary”**^28^*	Emotions/harms (motivation), Test design (opportunity)
	Amount of information	All	*“I think that just having one test is scary enough for someone, thinking they might have cancer. So just stick to that test, and when the results come back, then they can be told what the next step is.”**^28^*	Emotions (motivation), Health literacy (capability), Mass media (opportunity)
	Accurateness/thoroughness of the test	All	*"The day I had them both done, I think the nasal one, I think it missed something out" ^30^**^1^*	Benefits and harms (motivation)
	Trust in medical advice	All	*“If you've got to have a test, you've got to have a test”**^28^*	Primary care endorsement (opportunity)
	No sedative required	TNE, Cytosponge-TFF3	*"You can go straight home" ^30^**^1^*	Planning/transport (capability)
	Availability in GP's office	All	*"It's quicker, my doctor can do it and there's no messing around, no hospital appointments" ^28^*	Planning/self-efficacy (capability), Access to healthcare/location/patient navigation (opportunity)
	Cancer survival rates	Cytosponge-TFF3	*“Something so simple could help people get diagnosed earlier and increase the survival rates”**^31^*	Benefits and harms (motivation)
	Perceived costs	Cytosponge-TFF3	*"That's going to be an awful lot cheaper to do than an endoscopy at an hour a go with a gastroenterologist" ^28^*	Provider incentives (opportunity)
	Physician training	TNE	*[participants emphasized the requirement for adequately trained endoscopists to perform TNE and some expressed reservations over their general practitioners (GPs) taking up the role]**^30^*	Provider skills (opportunity)
Acting	Physical discomfort	All	*"Not so much with the nasal one but with the, with the oral one, it was very bad gagging reflex" ^30^*	Benefits and harms (motivation)
	Claustrophobic feelings	EGD	*[Feeling of being in a production line]**^30^*	Emotions/benefits and harms (motivation)
	Ability to speak with endoscopist and sit up	TNE	*“I could watch it on the monitor”**^30^*	Self-efficacy (capability), Test design (opportunity)
	Timing	All	*“Yeah, so what happens? Once it goes to the lab, how long will it take before you find out whether you've got it?”**^28^*	Planning (capability), Convenience/patient navigation (opportunity)

GORD, gastro-oesophageal reflux disease, BO, Barrett's oesophagus; OAC, oesophageal adenocarcinoma; EGD, conventional upper endoscopy; TNE, unsedated transnasal endoscopy; I-SAM, Integrated Screening Action Model.^17^ Authors’ interpretations are shown in brackets.

#### Preprocedural acceptability

##### Stage of awareness and engagement

Qualitative studies indicated that individuals did not have great awareness of oesophageal cancer risk, especially with regard to the link between GORD, BO and OAC (*perceived risk, knowledge*).^22^^,^^28^ Individuals’ awareness of the risks of OAC was positively affected by having relatives who had been diagnosed with oesophageal cancer (*emotions, perceived risk*).^31^ The effect of OAC awareness (i.e., awareness of symptoms, risk factors and screening options) on screening motivation has not been studied. In clinical trials, it seems that using phone calls to invite persons established a higher level of awareness and engagement (Table 3).^20^^,^^24^

**Table 3:** Uptake of OAC screening and determinants of uptake.

	Screening trial									Hypothetical screening		
Reference	Chak et al. (2014)^19^	Chang et al. (2011)^20^			Sami et al. (2015)^24^			Kadri et al. (2010)^29^	Fitzgerald et al. (2020)^27^	Gupta et al. (2014)^22^	Peters et al. A (2020)^34^	Peters et al. B (2020)^33^
Study design	RCT	Randomized pilot study			RCT			Prospective cohort study	RCT	survey	unlabelled dce	labelled dce
Setting	Outpatient clinic	Random sample Olmsted County (US) residents†			Random sample Olmsted County (US) residents†			12 general practices UK	109 general practices UK	Community sample MN	Population registry sample	Population registry sample
Population	45–85 y, veterans	>50 y, with GORD symptoms			> 50 y, with GORD symptoms			50–70 y, PPI records§	> 50 y, PPI records§	> 50 y, with GORD symptoms	50–74 y	50–74 y
Invitation approach	Mail/flyer, no reminder	Up to 3 phone calls			Up to 3 phone calls			Letter GP, no reminder	Letter GP, no reminder	NR	Postal mail	Postal mail
Timing eligibility screening interview	Before invite	Before invite			Before invite			After invite	After invite	NA	NA	NA
Incentive	$20	Not mentioned			Not mentioned			Not mentioned	Not mentioned	Not mentioned	Not mentioned	Not mentioned
Screening modality	TNE/ECE‡	TNE	ECE	EGD	huTNE	muTNE	EGD	Cytosponge-TFF3	Cytosponge-TFF3	Hypothetical	Hypothetical	Hypothetical
Invited, n	1210	52	52	81	151	158	150	2696	6983	136 filled out survey	375 filled out survey	554 filled out survey
Expressed interest in screening (%)	15·2	50·0	48·1	40·7	47·7	48·1	40·7	23·2	39·2	71·4	62·8	70·5
Completed procedure (%)	14·5	38·5	38·5	24·7	45·7	48·1	40·7	18·6	24·2			

Determinant*												
Increased age		NS			NS					NS	NS	–
Sex, male		+			NS					NS	+	NS
Ethnicity		NS									NS	NS
Education		NS			NS					NS		-||
Marital status					NS					NS	NS	NS
Employment status					+**					NS	NS	NS
Prior endoscopy		NS								+ *†	+	+
Participated in population-based cancer screening programs											+	NS
Upper GI symptoms		NS			+					+	+	NS
Comorbidity y		NS			NS						NS	NS
Cancer worries											+	NS
Knowing someone with OAC										NS	NS	NS
Personal history of cancer										NS	NS	NS

huTNE, in clinic unsedated transnasal endoscopy; muTNE, mobile-based unsedated transnasal endoscopy; EGD, conventional upper endoscopy; ECE, oesophageal capsule endoscopy; NS, not significant.

* Statistically significant results in multivariate analysis (generally logistic regressions) in the selected studies are indicated with a plus-symbol (facilitators) or minus-symbol (barriers).

† Subjects were previously (1988 to 2009) mailed validated gastrointestinal symptom questionnaires.

‡ Subjects were randomized after agreement to participate.

§ Or other prescribed acid-suppressant therapy.

¶ The exclusion of participants after measuring their interest may result in lower uptake numbers.

||College/university.

** Unemployment/homemaker.

*† Colonoscopy.

##### Deciding to act

The I-SAM suggests that the decision to act can be understood in terms of *motivation, capability* and *opportunity.*^17^ *Motivation* to participate in OAC screening is influenced by emotions, perceived risk, benefits and harms. Individuals in included studies marked fear of having a test that could result in a cancer diagnosis *(emotions, perceived risk).*^28^^,^^30^^,^^31^ Others expressed fear for the screening test itself and the potential side effects such as string detachment and oesophageal damage (*emotions, test design*).^28^^,^^31^ Studies emphasized the importance of belief in screening *benefit* for screening motivation, which is related to perceived accurateness and thoroughness of the screening test.^30^^,^^33^^,^^34^ Furthermore, one screening trial and two surveys have shown that individuals with GORD are more likely to participate in OAC screening, indicating that experiencing symptoms is an additional *motivator*.^22^^,^^24^^,^^34^

The decision to accept screening also depends on an individual's *capability* (i.e., cognitive and physical resources) to undertake the activities involved in undergoing screening.^17^ The need for a sedative and associated transportation problems were identified as barriers (*planning, transport*).^28^^,^^30^^,^^31^ When asked about the Cytosponge-TFF3, most individuals thought that the act of swallowing the capsule would not be problematic, although few were concerned about swallowing the string (*self-efficacy*).^28^^,^^31^

Finally, the *opportunity* to participate in screening is influenced by social and physical factors.^17^ Medical professionals’ advice prompted participation in a screening test (*primary care endorsement*).^30^ Furthermore, individuals emphasized the requirement for adequately trained physicians to perform the screening test (*provider skills*).^30^^,^^31^^,^^33^^,^^34^ Availability of a screening test in the general practitioners (GPs) office (*access to health care, location, patient navigation)* and perceived low costs (*provider incentives*) were identified as facilitators.^19^^,^^28^^,^^30^^,^^31^

DCE studies which aimed to evaluate which of these factors were most important in decision-making both reported test accuracy to be most influential.^33^^,^^34^ To illustrate this, although respondents preferred a non-invasive breath or blood test over endoscopic and ingestible sampling tests, this only applied if sensitivity and specificity were above 80%.^33^ Practical factors such as screening location were least influential.^34^

#### Procedural acceptability

Physical discomfort due to pain, gagging, vomiting, and choking was a frequently expressed barrier for OAC screening in qualitative studies (*harms*).^28^^,^^30^^,^^31^^,^^33^^,^^34^ In the following, we describe studies that quantitatively measured discomfort for several tests during OAC screening (appendix p 16). Patient-reported anxiety during an OAC screening test was low and decreased further in the weeks after the test (appendix p 17).^18^^,^^29^^,^^32^

##### Conventional upper endoscopy

Sedated upper endoscopy was well-tolerated by individuals in one study that reported a mean overall tolerability score of 0·4 (0–10 Likert scale, with 0=best and 10=worst).^18^ In another study in which endoscopy was performed unsedated, subjects reported a higher level of discomfort (mean score 2·9 [0–8 Likert scale, with 0=no discomfort and 8=very discomforting]).^32^

##### Ultrathin transnasal endoscopy

Contact with the root of the tongue is avoided with the transnasal approach, which is thought to decrease the gagging reflex to improve tolerability. Subject-reported tolerability was measured inconsistently across studies but appeared reasonable,^18^, ^19^, ^20^^,^^36^ for example a mean tolerability score of 2.2 (0–10 Likert scale, with 0=best and 10=worst).^18^ The ability to sit up, watch the procedure on a screen and speak with the endoscopist reduced psychological distress (*self-efficacy, test design*).^30^

##### Oesophageal capsule endoscopy

Studies on oesophageal capsule endoscopy showed good tolerability, with only 3% of participants reporting severe discomfort due to gagging.^19^ Capsule endoscopy was preferred over other endoscopic tests.^19^^,^^22^^,^^35^ Unfortunately, accuracy is limited due to the small number of total frames per centimetre that can be collected. A study investigating tethered capsule microendoscopy reported moderate patient-reported tolerability (mean score 1·9 [0–4 VAS, with 0=no discomfort and 4 = *a* lot of discomfort]).^21^^,^^26^ This relatively high discomfort score might be caused by the wire attached to the capsule, triggering the gag reflex.

##### Non-endoscopic cell collection

No study on the Cytosponge-TFF3 measured gagging scores but patient experience scores were good; a mean acceptability score of 9·0 (0–10 VAS, with 0=completely unacceptable and 10=completely acceptable)^27^ and mean experience score of 7.0 (0–10 VAS, with 0=worst experience and 10=best experience).^29^

##### Waiting time

Being informed about the duration of the procedure and the waiting time to receive results is important for individuals (*planning, patient navigation*).^28^ One study adopted the waiting trade-off method; the mean number of days a patient was willing to wait for results was 5.8 days for conventional upper endoscopy and 7.4 days for unsedated transnasal endoscopy (difference not statistically significant).^18^

### Uptake of screening

Uptake of OAC screening was reported in five studies,^19^^,^^20^^,^^24^^,^^27^^,^^29^ and varied from 14·5% to 48·1% (Table 3). All five screening trials provided reasons for non-participation: 31% - 62% of invited persons declined participation/did not reply.^19^^,^^20^^,^^24^^,^^27^^,^^29^ Additional reasons (all <20%) included ‘ineligible’, ‘missed appointment’, ‘deceased’, ‘moved’, or ‘transportation’. Intended participation measured with questionnaires was considerably higher (62·8% to 71·4%) than uptake in screening trials.^22^^,^^33^^,^^34^ In the labelled DCE, offering a breath or a blood test was associated with increased participation probability (+12·7% and +13·7%, respectively); whereas upper endoscopy, transnasal endoscopy and a cell collection device were associated with decreased participation probability (−16·1%, −12·2% and −6·6%, respectively).^33^ Only 2·7% – 4·5% of participants in DCE studies consistently chose never to be screened.^33^^,^^34^

Table 3 also shows determinants that were reported as potential facilitators or barriers for participation. Frequent GORD symptoms, male gender, and previous endoscopy experience (for other indications) were facilitators.^22^^,^^24^^,^^33^^,^^34^ Most potential determinants were not statistically significantly related to participation in OAC screening.

## Discussion

This systematic review suggests that offering a minimally invasive screening test to detect early OAC or its precursors may be an acceptable strategy from the general public's perspective. Once aware of the risk for OAC, some individuals were motivated to participate in OAC screening due to perceived cancer risk or trust in medical advice, while others had a negative attitude towards OAC screening due to fear of a cancer diagnosis, test-induced pain and gagging or inconvenient practicalities. Non-invasive screening tests such as blood or breath analysis tests are generally preferred, but individuals were willing to trade off comfort level for more accurate tests. Transnasal endoscopy was well-tolerated. The tolerability of capsule endoscopy was high, but this test lacks clinical utility. Median acceptability ratings for the Cytosponge-TFF3 test were high. Nonetheless, uptake of OAC screening in studies ranged from 14·5% to 48·1%.^19^^,^^20^^,^^24^^,^^27^^,^^29^

A previous review including three articles to inform the Canadian Task Force on Preventive Health Care on GORD patients’ values and preferences found that unwillingness to be screened was related to anxiety and fear of gagging.^37^ We found sixteen additional studies that included individuals regardless of GORD symptoms and/or were published recently. Our analysis of the additional studies shows, amongst other findings, that unawareness of the link between GORD symptoms, BO and OAC might influence screening motivation.^22^^,^^28^^,^^31^ Low awareness of OAC as a disease entity was previously reported in an Irish study; in which only 26 of 279 individuals (9·2%) in a population sample were aware of oesophageal cancer.^38^ We expect an even lower level of awareness amongst people with low socio-economic status (SES) because they are more likely to have low health literacy (i.e., difficulty to find, understand, evaluate and/or apply health information). Further studies should assess GORD, BO and OAC disease awareness and risk perception amongst citizens in high-risk countries to inform public health officers on health education needs.

The decision to be screened (first time or repeatedly) also appears to be entangled with perceptions of and experience with OAC screening tests. Although profiled as less-invasive, ultrathin transnasal endoscopy and the Cytosponge-TFF3 were not superior to sedated upper endoscopy with regard to patient-reported discomfort/acceptability. The latter observation is in line with a previous individual patient data meta-analysis (IPDMA) on the acceptability of the Cytosponge-TFF3.^39^ However, the IPDMA also showed that individuals still preferred the Cytosponge-TFF3 over sedated investigations, according to the authors because it is less time-consuming and more practical.^28^^,^^39^ Participants in DCE studies included in the current review expressed a clear preference for non-invasive tests, such as breath or blood analysis.^33^^,^^34^ An important caveat to the acceptability of breath or blood analysis tests is that individuals are willing to trade off comfort level for screening test accurateness,^33^ which is in line with a systematic review of discrete choice experiments on cancer screening in general.^40^ Unfortunately, test accuracy appears to be most compromised in non-invasive breath analysis techniques compared with other potential OAC screening tests.^41^, ^42^, ^43^ Breath analysis needs further validation and accuracy will need to be substantially improved before the public will accept it. In summary, minimally invasive OAC screening tests are generally well-tolerated but not optimal in terms of intrusiveness, accurateness, and practicality; screening tests therefore need further development before they are ready for implementation.

From a public health perspective, high uptake of a potential future screening program might positively impact the number of OAC cases that will be prevented or detected early, under the prerequisite that the strategy is proven to be effective. However, uptake of OAC screening in studies (14·5% to 48·1%) was found to be low compared with what has been achieved in population-based cancer screening programs in the European Union. In comparison, uptake of colorectal, breast, and cervical cancer screening is 49·5%, 60·2% and 50·7%, respectively, in combined data from 22 countries.^44^ Uptake might increase if OAC screening continues to move towards clinical implementation, as this generally results in more public awareness of screening options. This hypothesis is supported by the observation that participation in colorectal cancer screening programs has also shown an increasing trend from screening studies^45^ towards clinical implementation (especially in Finland, the Netherlands, Portugal, Sweden, the UK and the US).^44^^,^^46^ This increase is likely related to increased health education, media campaigns and focused public health efforts targeting inequality groups to increase awareness of cancer and screening options.^46^ To give an example of how increased uptake may be accomplished for OAC screening; an interim analysis of an ongoing study using Cytosponge-TFF3 testing in primary care incorporated a personalized invitation approach, as opposed to a written general invitation, and reported a relatively high uptake of 60·5%.^47^ Evidence on barriers and facilitators is currently scarce; the impact of non-significant determinants identified in this review therefore requires further examination in population-based studies.

By comparing our findings with the I-SAM we identified the following research directions that need further exploration to fully understand the public's perspective: the influence of SES, health literacy, social stigmas, access to health care, and involvement of the GP in making a decision on screening participation are unclear (Figure 3). Studying and engaging people with low SES is of particular importance for OAC screening considering the association between SES and OAC risk factors (central obesity, smoking and alcohol consumption).^48^ It is conceivable that communication about these risk factors will enhance social stigmas of screening, which also requires attention in studies. Furthermore, the fact that attendance of BO surveillance is influenced by health insurance status in the US^49^ indicates that it is essential to establish equal access to screening and surveillance for underserved populations if a screening program were to be introduced, for example through public funding and using affordable, simple and easily accessible tests.

The key strength of this review is the inclusion of qualitative, mixed-methods, and quantitative studies, thereby enabling a broad and integrated summary of existing evidence on OAC screening acceptability. Our synthesis provides the basis for future research. A limitation is the paucity of qualitative data extracted from 3 studies exploring the acceptability of ultrathin transnasal endoscopy and Cytosponge-TFF3 testing. It is doubtful whether thematic saturation was sufficiently accomplished for the various contexts in which OAC screening might be offered in the future. We recognize that the I-SAM with focus on OAC should be considered as preliminary, and that further studies are needed to support and/or refine it. It is currently unclear if the findings are generalizable to other countries, given that national cancer screening policies vary widely between countries. The heterogeneous methodological approaches between studies is another limitation. Seven studies used unvalidated and inconsistent instruments to measure patient-reported outcomes, and the five screening trials had variable recruitment approaches. Therefore, data from this review do not allow us to establish the preferred screening test to optimize acceptability and uptake of OAC screening.

In conclusion, offering a screening test to detect precursors of OAC may be an acceptable addition to current prevention and early detection strategies from the public's perspective. Individuals value accurate and non-invasive screening tests, although comparability of uptake and discomfort scores between available minimally invasive tests is limited. Our synthesis can inform researchers and policy makers by identifying research gaps and serve as a guide in designing a screening strategy which has appeal to the general public, thereby increasing informed participation in potential future OAC screening and improving OAC outcomes.

## Funding

This study was funded by the Netherlands Organization for Health Research and Development (ZonMw) under grant 555,004,206.

## Contributors

YP, LR, PS and MB were involved in conception of the work and acquisition of funding. JS, YP, LR, PS and MB designed the study and wrote the protocol. JS and KvdV did the study search, study selection, data extraction, and risk of bias assessment. YP, LR, PS and MB supervised all the steps in the review process. YP accessed and verified the underlying data. JS did the data analysis and created the figures. All authors interpreted the findings. JS drafted the manuscript and appendix. All authors contributed to revision of the manuscript.

## Data sharing

Study data are available on request to the corresponding author.

## Declaration of interests

PS is receiving unrestricted grants from Pentax (Japan), Norgine (UK), Motus GI (USA), MicroTech (China) and The eNose Company (Netherlands) and is in the advisory board of Motus GI (USA) and Boston Scientific (USA). All other authors declare no competing interests.

## Supplementary materials

Image, application 1

Image, application 2
