# Supplementary material for: Public acceptance and uptake of oesophageal adenocarcinoma screening strategies: A mixed-methods systematic review
Source: eClinicalMedicine. 2022 Apr 4;46:101367. doi: 10.1016/j.eclinm.2022.101367 (PMC8987366; doi:10.1016/j.eclinm.2022.101367)
Supplement: Supplementary file 1 [file mmc1.pdf]

Appendix:

1. Eligibility criteria 2

Table S1. Eligibility criteria 2

2. Search 3

2.1 Search strategies 3

2.2 List of review papers selected for hand-searching references and citations 9

Table S2. Studies excluded from systematic review 9

3. Critical appraisal results 12

Table S3. Threats to rigour of qualitative studies 12

Table S4. Quality assessment of the DCE studies included in the review according to the ISPOR quality checklist. 13

Table S5. Quality assessment based on STROBE guideline 14

3.1 Risk of bias summary 15

4. Additional outcome data 16

Table S6. Patient-reported physical distress related to OAC screening 16

Table S7. Patient-reported psychological distress related to OAC screening 17

Table S8. Additional patient-reported outcome data 18

Table S9. Summary patient-reported outcomes per screening test 20

5. References 21

1. Eligibility criteria

Table S1. Eligibility criteria

PICOS component	Eligibility criteria and considered components
Population*	• Individuals of the general population with or without risk factors for BE or EAC (such as male gender, white race, age > 50 years, obesity or any other risk factor as defined by the study authors)<br>• Patients with chronic gastro-oesophageal reflux disease (GERD)<br><br>Exclusion:<br>• Previous diagnosis of oesophageal cancer or precancerous lesions (Barrett's oesophagus or dysplasia)
Intervention	(Hypothetical) screening with the aim of early detection of EAC and associated (pre)cancerous lesions, using any kind of screening modality such as;<br>• Esophagogastroduodenoscopy<br>• Transnasal endoscopy<br>• Non-endoscopic cell collection devices<br>• Breath analysis<br><br>Exclusion:<br>• Screening aimed at detection of other conditions of the oesophagus, such as oesophageal squamous cell carcinoma.
Comparator	Not applicable
Outcomes	Outcome measures Q1:<br>• Participation in (pilot) EAC screening<br>• Intended participation determined by hypothetical EAC screening<br><br>Outcome measures Q2:<br>Acceptability of EAC screening, including but not limited to;<br>• Perceived threat (e.g. perceived susceptibility vs perceived seriousness)<br>• Perceived benefits and harms<br>• Willingness to undergo the procedure initially and willingness to undergo the procedure again afterwards<br>• Cognitive, affective, physical and social factors considered in decision to be screened<br>• Preferred screening technique<br>• Tolerability of screening techniques
Study design	• Original studies<br>• Studies in English or Dutch language<br>• Peer-reviewed articles<br>• No limit on publication year<br><br>Exclusion:<br>• Conference abstracts or case reports<br>• Systematic review or meta-analysis (these were used for citation searching)

* For the preferred screening techniques and tolerability of screening techniques outcome, participants from control groups of studies investigating Barrett's oesophagus patients were included in case they met the in- and exclusion criteria.

2. Search

2.1 Search strategies

Ovid MEDLINE(R) ALL 1946 to December 02, 2020

1. Esophageal Neoplasms/

2. exp Esophagus/ and exp Neoplasms/

3. ((esophag$ or oesophag$) adj3 (neoplas$ or cancer$ or tumo?r or carcinoma$ or malignan$ or oncolog$ or adenocarcinoma$ or adeno-carcinoma$)).tw,kw.

4. Barrett Esophagus/

5. (Barret$ adj1 (esophag$ or oesophag$ or epitheli$ or metaplasi$ or syndrome?)).tw,kw.

6. ((esophag$ or oesophag$) adj3 (dysplasia or dysplastic$ or precancer$ or pre-cancer$ or premalignant$ or pre-malignan$)).tw,kw.

7. 1 or 2 or 3 or 4 or 5 or 6

8. Mass screening/

9. Early detection of cancer/

10. Gastroscopy/

11. Esophagoscopy/

12. Endoscopy/

13. Endoscopy, Gastrointestinal/

14. Capsule Endoscopy/

15. Conscious Sedation/

16. Diagnostic Techniques, Digestive System/

17. Cytodiagnosis/

18. (screening$ or screened or screen).tw,kw.

19. (early adj3 detect$).tw,kw.

20. (cytosponge$ or breath analy$ or endoscopy).tw,kw.

21. 8 or 9 or 10 or 11 or 12 or 13 or 14 or 15 or 16 or 17 or 18 or 19 or 20

22. health knowledge, attitudes, practice/

23. Decision Making/

24. exp "Patient Acceptance of Health Care"/

25. patient reported outcome measures/

26. exp patient satisfaction/ or patient preference/

27. Public Opinion/

28. (acceptab$ or anxiety or acceptance or attitude$ or barrier$ or belief$ or choice or decide$ or decision$ or discomfort or opinion$ or patient perspective$ or perception$ or (perceive$ adj2 (threat$ or risk$)) or prefer$ or self-efficacy or tolerability or willingness or worrie$ or worry$).tw,kw.

29. 22 or 23 or 24 or 25 or 26 or 27 or 28

30. exp community participation/ or patient participation/

31. (participat$ or ((predict$ or expect$ or determine$ or anticipat$) adj 3 uptake)).tw,kw.

32. 30 or 31

33. Qualitative research/

34. Interview, Psychological/ or Interview/

35. Focus groups/

36. Health Care Surveys/

37. Questionnaires/

38. ((semi-structured or semistructured or unstructured or informal or in-depth or indepth or structure$ or guide$) and (interview$ or discussion$ or question?aire$)).tw,kw.

39. 33 or 34 or 35 or 36 or 37 or 38

40. 29 or 32 or 39

41. 7 and 21 and 40

Hits: 1610

Additional hits update dd 2 July 2021: 58

Embase 1974 to 2020 December 02

1. esophagus tumor/ or esophagus cancer/

2. exp Esophagus/ and exp Neoplasm/

3. ((esophag$ or oesophag$) adj3 (neoplas$ or cancer$ or tumo?r or carcinoma$ or malignan$ or oncolog$ or adenocarcinoma$ or adeno-carcinoma$)).tw,kw.

4. Barrett Esophagus/

5. (Barret$ adj1 (esophag$ or oesophag$ or epitheli$ or metaplasi$ or syndrome?)).tw,kw.

6. ((esophag$ or oesophag$) adj3 (dysplasia or dysplastic$ or precancer$ or pre-cancer$ or premalignant$ or pre-malignan$)).tw,kw.

7. 1 or 2 or 3 or 4 or 5 or 6

8. Mass screening/

9. early cancer diagnosis/

10. gastrointestinal endoscopy/ or gastroscopy/

11. esophagoscopy/

12. exp capsule endoscopy/

13. cancer cytodiagnosis/ or cytodiagnosis/

14. conscious Sedation/

15. (screening$ or screened or screen).tw,kw.

16. (early adj3 detect$).tw,kw.

17. (cytosponge$ or breath analy$ or endoscopy).tw,kw.

18. 8 or 9 or 10 or 11 or 12 or 13 or 14 or 15 or 16 or 17

19. health knowledge, attitudes, practice/

20. decision Making/

21. exp "Patient Acceptance of Health Care"/

22. patient-reported outcome/

23. exp patient satisfaction/ or patient preference/

24. Public Opinion/

25. (acceptab$ or anxiety or acceptance or attitude$ or barrier$ or belief$ or choice or decide$ or decision$ or discomfort or opinion$ or patient perspective$ or perception$ or (perceive$ adj2 (threat$ or risk$)) or prefer$ or self-efficacy or tolerability or willingness or worrie$ or worry$).tw,kw.

26. 19 or 20 or 21 or 22 or 23 or 24 or 25

27. exp community participation/ or patient participation/

28. (participation or ((predict$ or expect$ or determine$ or anticipat$) adj 3 uptake)).tw,kw.

29. 27 or 28

30. Qualitative research/

31. Interview, Psychological/ or Interview/

32. Focus groups/

33. Health Care Surveys/

34. Questionnaires/

35. ((semi-structured or semistructured or unstructured or informal or in-depth or indepth or structure$ or guide$) and (interview$ or discussion$ or question?aire$)).tw,kw.

36. 30 or 31 or 32 or 33 or 34 or 35

37. 26 or 29 or 36

38. 7 and 18 and 37

39. limit 38 to conference abstract

40. 38 not 39

Hits: 1895

Additional hits update dd 2 July 2021: 99

APA PsycInfo 1806 to 2020 week 1

1. exp Esophagus/ and exp Neoplasms/

2. ((esophag$ or oesophag$) adj3 (neoplas$ or cancer$ or tumo?r or carcinoma$ or malignan$ or oncolog$ or adenocarcinoma$ or adeno-carcinoma$)).mp.

3. (Barret$ adj1 (esophag$ or oesophag$ or epitheli$ or metaplasi$ or syndrome?)).mp.

4. ((esophag$ or oesophag$) adj3 (dysplasia or dysplastic$ or precancer$ or pre-cancer$ or premalignant$ or pre-malignan$)).mp.

5. 1 or 2 or 3 or 4

6. screening/

7. cancer screening/

8. (screening$ or screened or screen).mp.

9. (early adj3 detect$).mp.

10. (breath analy$ or (capsule adj1 endoscopy) or (conscious adj1 sedation) or cytosponge$ or cytodiagnos$ or endoscop$ or esophagoscop$ or gastroscop$).mp.

11. 6 or 7 or 8 or 9 or 10

12. choice behavior/

13. Decision Making/

14. exp health attitudes/

15. exp Social Acceptance/

16. patient reported outcome measures/

17. exp preferences/ or patient preference/

18. Public Opinion/

19. exp participation/

20. exp perception/

21. "susceptibility (disorders)"/

22. (acceptab$ or anxiety or acceptance or attitude$ or barrier$ or belief$ or choice or decide$ or decision$ or discomfort or opinion$ or participat$ or patient perspective$ or perception$ or (perceive$ adj2 (threat$ or risk$)) or prefer$ or self-efficacy or tolerability or willingness or worrie$ or worry$ or uptake).mp.

23. 12 or 13 or 14 or 15 or 16 or 17 or 18 or 19 or 20 or 21 or 22

24. Qualitative research/

25. interviews/ or focus group interview/ or psychodiagnostic interview/ or semi-structured interview/

26. focus group/

27. surveys/

28. Questionnaires/

29. ((semi-structured or semistructured or unstructured or informal or in-depth or indepth or structure$ or guide$) and (interview$ or discussion$ or question?aire$)).mp.

30. 24 or 25 or 26 or 27 or 28 or 29

31. 23 or 30

32. 5 and 11 and 31

Hits: 12

Additional hits update dd 2 July 2021: 0

2.2 List of review papers selected for hand-searching references and citations

1. Hamel C, Ahmadzai N, Beck A, et al. Screening for esophageal adenocarcinoma and precancerous conditions (dysplasia and Barrett's esophagus) in patients with chronic gastroesophageal reflux disease with or without other risk factors: two systematic reviews and one overview of reviews to inform a guideline of the Canadian Task Force on Preventive Health Care (CTFPHC). Syst Rev 2020; 9(1): 20.

2. Iqbal U, Siddique O, Ovalle A, Anwar H, Moss SF. Safety and efficacy of a minimally invasive cell sampling device ('Cytosponge') in the diagnosis of esophageal pathology: A systematic review. European Journal of Gastroenterology and Hepatology 2018; 30(11): 1261-9.

3. Januszewicz W, Tan WK, Lehovsky K, et al. Safety and Acceptability of Esophageal Cytosponge Cell Collection Device in a Pooled Analysis of Data From Individual Patients. Clinical Gastroenterology and Hepatology 2019; 17(4): 647-56.e1.

4. Sami SS, Subramanian V, Ortiz-Fernandez-Sordo J, et al. Performance characteristics of unsedated ultrathin video endoscopy in the assessment of the upper GI tract: systematic review and meta-analysis. Gastrointest Endosc 2015; 82(5): 782-92.

Table S2. Studies excluded from systematic review

	Study	Reason for exclusion
	**Electronic database searching**	
1	Abou-Nader L, Wilson JA, Paleri V. Transnasal oesophagoscopy: Diagnostic and management outcomes in a prospective cohort of 257 consecutive cases and practice implications. Clin Otolaryngol 2014; 39(2): 108-13.	Indication for intervention broader than OAC screening
2	Beg S, Card T, Warburton S, et al. Diagnosis of Barrett's esophagus and esophageal varices using a magnetically assisted capsule endoscopy system. Gastrointestinal Endoscopy 2020; 91(4): 773-81.e1.	Did not stratify results for BE and population of interest
3	Birkner B, Fritz N, Schatke W, Hasford J. A prospective randomied comparison of unsedated ultrathin versus standard esophagogastroduodenoscopy in routine outpatient gastroenterology practice: Does it work better through the nose? Endoscopy 2003; 35(8): 647-51.	Indication for intervention broader than OAC screening
4	Catanzaro A, Faulx A, Isenberg GA, et al. Prospective evaluation of 4-mm diameter endoscopes for esophagoscopy in sedated and unsedated patients. Gastrointest Endosc 2003; 57(3): 300-4.	Indication for intervention broader than OAC screening
5	De Jonge PJF, Van Eijck BC, Geldof H, et al. Capsule endoscopy for the detection of oesophageal mucosal disorders: A comparison of two different ingestion protocols. Scandinavian Journal of Gastroenterology 2008; 43(7): 870-7.	Did not stratify results for BE and population of interest
6	Duvvuri A, Desai M, Vennelaganti S, et al. Diagnostic accuracy of a novel third generation esophageal capsule as a non-invasive detection method for Barrett's esophagus: A pilot study. Journal of Gastroenterology and Hepatology 2020.	Had only participants with BE
7	Guo L, Zhang S, Liu S, et al. Determinants of participation and detection rate of upper gastrointestinal cancer from population-based screening program in China. Cancer Medicine 2019; 8(16): 7098-107.	Screening for oesophageal squamous cell carcinoma
8	Iyer PG, Taylor WR, Johnson ML, et al. Highly Discriminant Methylated DNA Markers for the Non-endoscopic Detection of Barrett's Esophagus. American Journal of Gastroenterology 2018; 113(8): 1156-66.	Did not stratify results for BE and population of interest
9	Jobe BA, Hunter JG, Chang EY, et al. Office-based unsedated small-caliber endoscopy is equivalent to conventional sedated endoscopy in screening and surveillance for Barrett's esophagus: A randomized and blinded comparison. American Journal of Gastroenterology 2006; 101(12): 2693-703.	Did not stratify results for BE and population of interest

10	Kang D, Lim CH, Choi MG, et al. An Operable, Portable, and Disposable Ultrathin Endoscope for Evaluation of the Upper Gastrointestinal Tract. Dig Dis Sci 2019; 64(7): 1901-7.	Indication for intervention broader than OAC screening
11	Kramer JR, Arney J, Chen J, et al. Patient-centered, comparative effectiveness of esophageal cancer screening: protocol for a comparative effectiveness research study to inform guidelines for evidence-based approach to screening and surveillance endoscopy. BMC health services research 2012; 12: 288.	Study protocol
12	Liao Z, Gao R, Xu C, Xu DF, Li ZS. Sleeve string capsule endoscopy for real-time viewing of the esophagus: a pilot study (with video). Gastrointestinal Endoscopy 2009; 70(2): 201-9.	Screening for oesophageal squamous cell carcinoma
13	Marelli L, Jaboli FM, Jackson L, et al. A pilot study comparing ESO-2 capsule endoscopy with conventional upper endoscopy for the assessment of uncomplicated heartburn and dyspepsia. Frontline Gastroenterology 2013; 4(2): 96-101.	Indication for intervention broader than OAC screening
14	Preiss C, Charton JP, Schumacher B, Neuhaus H. A randomized trial of unsedated transnasal small-caliber esophagogastroduodenoscopy (EGD) versus peroral small-caliber EGD versus conventional EGD. Endoscopy 2003; 35(8): 641-6.	Indication for intervention broader than OAC screening
15	Qiu L, Chuttani R, Zhang S, et al. Diagnostic imaging of esophageal epithelium with clinical endoscopic polarized scanning spectroscopy instrument. Conference proceedings : 2009; Annual International Conference of the IEEE Engineering in Medicine and Biology Society. IEEE Engineering in Medicine and Biology Society. Conference. 2009: 1997-2000.	No outcome of interest
16	Ramirez FC, Shaukat MS, Young MA, Johnson DA, Akins R. Feasibility and safety of string, wireless capsule endoscopy in the diagnosis of Barrett's esophagus. Gastrointestinal Endoscopy 2005; 61(6): 741-6.	Had only participants with BE
17	Ross-Innes CS, Debiram-Beecham I, O'Donovan M, et al. Evaluation of a minimally invasive cell sampling device coupled with assessment of trefoil factor 3 expression for diagnosing Barrett's esophagus: a multi-center case-control study. PLoS Med 2015; 12(1): e1001780.	Did not stratify results for BE and population of interest
18	Saidi F, Malekzadeh R, Sotoudeh M, et al. Endoscopic esophageal cancer survey in the western part of the Caspian Littoral. Diseases of the Esophagus 2002; 15(3): 214-8.	No outcome of interest
19	Sami SS, Iyer PG, Pophali P, et al. Acceptability, Accuracy, and Safety of Disposable Transnasal Capsule Endoscopy for Barrett's Esophagus Screening. Clinical Gastroenterology and Hepatology 2019; 17(4): 638-46.e1.	Did not stratify results for BE and population of interest
20	Schuldt AL, Kirsten H, Tuennemann J, et al. Necessity of transnasal gastroscopy in routine diagnostics: A patient-centred requirement analysis. BMJ Open Gastroenterology 2019; 6(1).	Indication for intervention broader than OAC screening
21	Shariff MK, Bird-Lieberman EL, O'Donovan M, et al. Randomized crossover study comparing efficacy of transnasal endoscopy with that of standard endoscopy to detect Barrett's esophagus. Gastrointest Endosc 2012; 75(5): 954-61.	Did not stratify results for BE and population of interest
22	Shariff MK, Varghese S, O'Donovan M, et al. Pilot randomized crossover study comparing the efficacy of transnasal disposable endosheath with standard endoscopy to detect Barrett's esophagus. Endoscopy 2016; 48(2): 110-6.	Did not stratify results for BE and population of interest
23	Thota PN, Zuccaro G, Vargo JJ, Conwell DL, Dumot JA, Xu M. A randomized prospective trial comparing unsedated esophagoscopy via transnasal and transoral routes using a 4-mm video endoscope with conventional endoscopy with sedation. Endoscopy 2005; 37(6): 559-65.	Indication for intervention broader than OAC screening
24	Xiao HF, Yan SP, Chen YF, et al. Community-based upper gastrointestinal cancer screening in a randomized controlled trial: Baseline results in a non-high-incidence area. Cancer Prevention Research 2020; 13(3): 317-27.	Screening for oesophageal squamous cell carcinoma
25	Yamasaki Y, Takenaka R, Hori K, et al. Tolerability of magnifying narrow band imaging endoscopy for esophageal cancer screening. World Journal of Gastroenterology 2015; 21(9): 2793-9.	Screening for oesophageal squamous cell carcinoma
26	Yang H, Berner A, Mei Q, et al. Cytologic screening for esophageal cancer in a high-risk population in Anyang County, China. Acta Cytologica 2002; 46(3): 445-52.	Screening for oesophageal squamous cell carcinoma
27	Zhu J, Zhou Y, Ma S, et al. The association between anxiety and esophageal cancer: A nationwide population-based study. Psycho Oncology 2020.	Screening for oesophageal squamous cell carcinoma
	**Updated search**	
28	Shaheen NJ, Komanduri S, Muthusamy VR, et al. Acceptability and Adequacy of a Non-endoscopic Cell Collection Device for Diagnosis of Barrett's Esophagus: Lessons Learned. Dig Dis Sci 2021.	Did not stratify results for BE and population of interest
	**Hand-searching of references and citations**	
29	Alexandridis, E. G., Trimble, K., Hayes, P., & Plevris, J. N. Randomized Prospective Trial of TransNasal Versus Standard Upper Diagnostic Endoscopy Under Local Anaesthetic: Interim Analysis of Endoscopy Quality, Patient Acceptability and Tolerability. Gastrointestinal Endoscopy 2012, 75(4), AB283.	Indication for intervention broader than OAC screening
30	Chan DK, Zakko L, Visrodia KH, et al. Breath Testing for Barrett's Esophagus Using Exhaled Volatile Organic Compound Profiling With an Electronic Nose Device. Gastroenterology 2017; 152(1): 24-6.	No outcome of interest

31	Faulx AL, Catanzaro A, Zyzanski S, et al. Patient tolerance and acceptance of unsedated ultrathin esophagoscopy. Gastrointest Endosc 2002; 55(6): 620-3.	Indication for intervention broader than OAC screening
32	Horiuchi A, Nakayama Y, Hidaka N, Ichise Y, Kajiyama M, Tanaka N. Prospective comparison between sedated high-definition oral and unsedated ultrathin transnasal esophagogastroduodenoscopy in the same subjects: pilot study. Dig Endosc 2009; 21(1): 24-8.	Indication for intervention broader than OAC screening
33	Lao-Sirieix P, Rous B, O'Donovan M, Hardwick RH, Debiram I, Fitzgerald RC. Non-endoscopic immunocytological screening test for Barrett's oesophagus. Gut 2007; 56(7): 1033-4.	Indication for intervention broader than OAC screening
34	Murata A, Akahoshi K, Sumida Y, Yamamoto H, Nakamura K, Nawata H. Prospective randomized trial of transnasal versus peroral endoscopy using an ultrathin videoendoscope in unsedated patients. J Gastroenterol Hepatol 2007; 22(4): 482-5.	Indication for intervention broader than OAC screening
35	Peters Y, Schrauwen RWM, Tan AC, Bogers SK, de Jong B, Siersema PD. Detection of Barrett's oesophagus through exhaled breath using an electronic nose device. Gut 2020; 69(7): 1169-72.	Did not stratify results for BE and population of interest
36	Saeian K, Staff DM, Vasilopoulos S, et al. Unsedated transnasal endoscopy accurately detects Barrett's metaplasia and dysplasia. Gastrointest Endosc 2002; 56(4): 472-8.	Did not stratify results for BE and population of interest
37	Stroppa I, Grasso E, Paoluzi OA, et al. Unsedated transnasal versus transoral sedated upper gastrointestinal endoscopy: a one-series prospective study on safety and patient acceptability. Dig Liver Dis 2008; 40(9): 767-75.	Indication for intervention broader than OAC screening
38	Tatsumi Y, Harada A, Matsumoto T, Tani T, Nishida H. Feasibility and tolerance of 2-way and 4-way angulation videoscopes for unsedated patients undergoing transnasal EGD in GI cancer screening. Gastrointest Endosc 2008; 67(7): 1021-7.	Gastric cancer screening
39	Wildi SM, Wallace MB, Glenn TF, Mokhashi MS, Kim CY, Hawes RH. Accuracy of esophagoscopy performed by a non-physician endoscopist with a 4-mm diameter battery-powered endoscope. Gastrointest Endosc 2003; 57(3): 305-10.	Indication for intervention broader than OAC screening
40	Yagi J, Adachi K, Arima N, et al. A prospective randomized comparative study on the safety and tolerability of transnasal esophagogastroduodenoscopy. Endoscopy 2005; 37(12): 1226-31.	Indication for intervention broader than OAC screening
41	Zaman A, Hahn M, Hapke R, Knigge K, Fennerty MB, Katon RM. A randomized trial of peroral versus transnasal unsedated endoscopy using an ultrathin videoendoscope. Gastrointest Endosc 1999; 49(3 Pt 1): 279-84.	Indication for intervention broader than OAC screening
42	Zaman A, Hapke R, Sahagun G, Katon RM. Unsedated peroral endoscopy with a video ultrathin endoscope: patient acceptance, tolerance, and diagnostic accuracy. Am J Gastroenterol 1998; 93(8): 1260-3.	Indication for intervention broader than OAC screening

3. Critical appraisal results

Table S3. Threats to rigour of qualitative studies

	Answer options			
	Low quality	Low quality	Medium quality	High quality
**Quality appraisal question**	**Not at all/not stated**	**Few steps**	**Several steps**	**A thorough attempt**
1. Were steps taken to increase rigour in sampling?	Tan et al (2019)[3]		Freeman et al (2017)[4]	McGoran et al (2019)[5]
2. Were steps taken to increase rigour in data collection?	Tan et al (2019)[3]			McGoran et al (2019)[5]<br>Freeman et al (2017)[4]
3. Were steps taken to increase rigour in data analysis?			Tan et al (2019)[3]<br>Freeman et al (2017)[4]	McGoran et al (2019)[5]
	**No grounding**	**Limited grounding/support**	**Fairly well grounded**	**Well-grounded/supported**
4. Were the findings of the study grounded in/supported by data?				Tan et al (2019)[3]<br>McGoran et al (2019)[5]<br>Freeman et al (2017)[4]
	**Limited breadth and depth**	**Good/fair breadth, limited depth**	**Good/fair depth, limited breadth**	**Good/fair breadth and depth**
5. Breadth and depth of findings?		Tan et al (2019)[3]	McGoran et al (2019)[5]<br>Freeman et al (2017)[4]	
	**Not at all**	**A little**	**Somewhat**	**A lot**
6. To what extent did the study privilege the perspectives and experiences			Tan et al (2019)[3]<br>Freeman et al (2017)[4]	McGoran et al (2019)[5]

Table S4. Quality assessment of the DCE studies included in the review according to the ISPOR quality checklist.

	**Reference**	**Peters et al A (2020)[6]**	**Peters et al B (2020)[7]**
**1.**	**Research question**		
	Well-defined research question and hypothesis	Yes	Yes
	Decision-making or policy context	Yes	Yes
	Rationale for using conjoint analysis	Yes	Yes
**2.**	**Attributes and levels**		
	Literature/clinical trials used to attribute selection	Yes	Yes
	Focus groups (Pts/MDs) used for attribute selection	Yes	Yes
	Level selection for each attribute justified	No	Yes
**3.**	**Construction of tasks**		
	Number of attributes in each task justified	Yes	Yes
	Opt-out included	Yes	Yes
**4.**	**Experimental design**		
	Design justified	Yes	Yes
	Properties of experimental design evaluated	Yes	Yes
	Number of conjoint tasks appropriate	Yes	Yes
**5.**	**Preference elicitation**		
	Sufficient motivation and explanation for tasks	Yes	Yes
	Appropriate elicitation format	Yes	Yes
	Additional qualifying questions	No	No
**6.**	**Instrument design**		
	Respondent information collected	Yes	Yes
	Definition attributes and levels	Yes	Yes
	Burden of instrument appropriate	Yes	Yes
**7.**	**Data-collection plan**		
	Sampling strategy	Yes	Yes
	Mode of administration	Yes	Yes
	Ethical considerations	No	Yes
**8.**	**Statistical analyses**		
	Respondent characteristics examined	Yes	Yes
	Rationality, validity, reliability of responses examined	Yes	Yes
	Model estimation appropriate	Yes	Yes
**9.**	**Results and conclusions**		
	Study results reflected testable hypothesis	Yes	Yes
	Study conclusions supported by evidence	Yes	Yes
	Limitations and generalizability discussed	Yes	Yes
**10.**	**Study presentation**		
	Study importance	Yes	Yes
	Description methods	Yes	Yes
	Implications	Yes	Yes

Table S5. Quality assessment based on STROBE guideline

	Eliakam et al (2004)[8]	Wilkins et al (2005)[9]	Essink et al (2007)[10]	Ramirez et al (2008)[11]	Kadri et al (2010)[12]	Chang et al (2011)[13]	Mori et al (2011)[14]	Peery et al (2012)[15]	Chak et al (2014)[16]	Sami et al (2015)[17]	Gora et al (2016)[18]	Blevins et al (2018)[19]	Fitzgerald et al (2020)[20]
**Construct assessed**	Preferred test	Willingness	Tolerability, anxiety	Tolerability, preferred test	Uptake, acceptability, anxiety	Uptake, tolerability	Tolerability	Tolerability, anxiety	Uptake, tolerability	Uptake, tolerability	Tolerability, anxiety	Tolerability	Uptake, acceptability
**Reporting**													
1 Was the target population specified?	1	1	1	1	1	1	1	1	1	1	1	1	1
2 Was the sample size adequate?	0	1	1	1	1	1	1	1	1	1	0	1	1
3 Was information given on non-responders?	0	1	0	0	1	1	0	0	1	1	0	0	1
4 Were known and validated instruments used for measurement?	0	0	1	0	1	0	1	0	0	0	0	0	1
5 Were inclusion criteria specified?	1	1	1	0	1	1	1	1	1	1	1	1	1
6 Was information on persons actually studied reported in detail?	1	1	1	1	1	1	1	1	1	1	1	1	1
7 Were confidence intervals or standard errors presented for the estimates of prevalence?	0	0	1	1	1	1	1	1	0	1	1	1	1
8 Was there an IRB in the study?	1	1	1	1	1	0	1	1	1	1	1	1	1
**Score**	**4**	**6**	**7**	**5**	**8**	**6**	**7**	**6**	**6**	**7**	**5**	**6**	**8**

3.1 Risk of bias summary

Risk of bias in qualitative studies

Overall study quality was medium to high for 2 included qualitative studies,[4,5] and medium for 1 included qualitative study.[3] Breadth of findings was limited in all 3 qualitative studies to answer the review question, as they were conducted in the context of a specific screening test instead of exploring acceptability of BE/EAC screening in general. One study was judged as making 'no steps' to increasing rigor in sampling and data collection because this study analysed Facebook comments posted by random Facebook users.[3]

Risk of bias in DCE studies

The ISPOR checklist is not designed to produce a quality score based on study characteristics, but instead is intended as a means to highlight methodological aspects and their reporting.[21]

Both DCE studies were authored by 2 members of the review team. Assessment of these papers was carried out by other members of the team. Both DCE studies included information on the rationale for undertaking a DCE, the construction of tasks, preference elicitation (i.e., the way in which preference questions were framed) and discussion around the implications of the study findings. DCE study designs were supported by best practice, with both studies stating that they used a combination of literature and focus groups to select attributes and levels.[6,7] Both DCE studies conducted sensitivity analyses by removing participants with task nonattendance or when failing dominance tests.[6,7]

Risk of bias in studies measuring patient-reported outcomes

Since there is no standard tool available to assess the risk of bias for patient-reported outcome measures in prospective studies, we used the adapted STROBE checklist previously published by Chad-Friedman et al.[22] The adapted checklist consists of 8 domains for assessing bias. Each domain was coded as 0 (no) or 1 (yes), with total risk-of-bias scores ranging from 0 (highest risk) to 8 (lowest possible risk). Main study shortcomings for measuring patient-reported outcomes were a lack of information on non-responders and using unvalidated instruments.

4. Additional outcome data

Table S6. Patient-reported physical distress related to OAC screening

Reference	Instrument	Scoring Instructions and Cutoff Score	Screening test	Timing: before or after screening	Overall discomfort score: %/M, ± SD/SEM/ range/IQR	Pain score: %/M, ± SD/SEM/ range/IQR	Choking score: %/M, ± SD/SEM/ range/IQR/%	Gagging score: %/M, ± SD/SEM/ range/IQR/%	Tolerability score: interpretation
Blevins et al (2018)[19]	Ten-point scale	1=not specified, 10=least tolerable	huTNE	24h after	2.2 (SEM, 0.3)	2.8 (SEM, 0.3)	0.8 (SEM, 0.2)	1.1 (SEM, 0.3)	+
			muTNE	24h after	1.9 (SEM, 0.1)	2 (SEM, 0.2)	0.6 (SEM, 0.2)	1.3 (SEM, 0.3)	+
			EGD (sedated)	24h after	0.4 (SEM, 0.1)	0.1 (SEM, 0.1)	0 (SEM, 0)	0.2 (SEM, 0.1)	++
Chak et al (2014)[16]	Ten-point scale	1=minimal discomfort, 10=severe discomfort, expressed in (%) crossing 3	TNE	Directly after	12.6%	22%	11.5%	30%	+
			ECE	Directly after	0%	1.1%	0%	3.3%	++
Chang et al (2011)[13]	Eleven-point Likert scale	0=best experience, 10= worst experience	TNE	NR	3.5 (range, 0-9)	1.0 (range, 0-5)	0 (range, 0-7)	0.5 (range, 0-9)	+
Essink et al (2007)[10]	Nine-point Likert scale	0=no discomfort, 8=very discomforting	EGD (9% sedated)	Measured during insertion, procedure, removing, directly after and pooled	2.9 (SD, 2.4)	0.9 (SD, 1.8)	..	..	+/-
Gora et al (2016)[18]	Four-point Likert scale	1=no discomfort, 4=a lot of discomfort	TCE	After	1.9 (IQR, 0.9)	..	..		+/-
Mori et al (2011)[14]	Eleven-point VAS	0=no gagging, 10= unbearable gagging	TNE	After	..	..	..	2 (IQR, 0–4)	++
			UUE	After	..	..	..	4 (IQR, 2–6)	+
			EGD (sedated)	After	..	..	..	4 (IQR, 2–6)	+
Peery et al (2012)[15]	Ten-point Likert scale	1=well-tolerated, 10= poorly tolerated	TNE	During	1 (IQR, 2)	2 (IQR, 2)	1 (IQR, 1)	2 (IQR, 2)	++
Ramirez et al (2008)[11]	Four-point Likert scale	0=no discomfort, 3= severe, expressed in % reporting no or mild; % reporting severe	ECE	Directly after	79%; 1%	..	..	75%; 3%	+
Wilkins et al (2005)[9]	..	Measured as % completing the procedure	UUE	..	94·6%	..	..	..	NA

huTNE, in clinic unsedated transnasal endoscopy; *muTNE*, mobile-based unsedated transnasal endoscopy; *EGD*, conventional upper endoscopy; *ECE*, esophageal capsule endoscopy; *TCE*, tethered capsule endomicroscopy; *UUE*, unsedated ultrathin oral endoscopy.

Table S7. Patient-reported psychological distress related to OAC screening

Reference	Validated measure	Unvalidated measure	Scoring Instructions and Cutoff Score	Construct	Screening test	Timing: before or after screening	Score: M ± SD/SEM/ range/IQR	Anxiety score: interpretation
Blevins et al (2018)[19]	NA	Ten-point Likert scale	1=minimal, 10=maximal	Anxiety	huTNE	24h after	2.2 (SEM, 0.3)	-
					muTNE	24h after	2.7 (SEM, 0.3)	-
					EGD	24h after	0.9 (SEM, 0.2)	-/-
Chak et al (2014)[16]	NA	Ten-point Likert scale	0=minimal, 10=severe, expressed in (%) crossing 3	Anxiety	TNE	1. Before	38%	+/-
						2. During	33%	+/-
					ECE	1. Before	17%	-
						2. During	16%	-
Chang et al (2011)[13]	NA	Eleven-point Likert scale	0=best experience, 10=worst experience	Anxiety	TNE	NR	1.5 (range, 0-7)	-
Essink et al (2007)[10]	1. HAD	NA	0=low, 21=high, > 11=clinically significant	Anxiety	EGD	1. Baseline	6.9	-
						2. Endoscopy day	6.5	-
						3. 1 week after	5.3	-
						4. 1 month after	5.2	-
	2. Impact of events scale	NA	0-35 p for intrusive thoughts + 0-40 p for avoidance thoughts = 75p (>26 high test induced stress)	Intrusive and avoiding thoughts regarding the endoscopy	EGD	1. Baseline	12.9 (14.7)	
				regarding the communication of the test result		2. 1 week after	9.4 (14.3)	
Gora et al (2016)[18]	NA	Five-point Likert scale	1=not at all anxious, 5=extremely anxious	Anxiety	TCE	Before	2.1 (0.8)	-
Kadri et al (2010)[12]	1. STAI		>40 clinically significant	Anxiety	Cytosponge-TFF3	1. 30 min after	33.1 (IQR, 26.6-43.3)	-
						2. 7 days after	30.0 (IQR, 20.0-40.0);	-
						3. 90 days after	26.6 (IQR, 20.0-36.6)	-
	2. Impact of events scale	NA	0-35 p for intrusive thoughts + 0-40 p for avoidance thoughts = 75p (< 8.5 low stress; > 19 high test induced stress)	Distress	Cytosponge-TFF3	1. 7 days after	16	Intermediate test-induced distress

Peery et al (2012)[15]	Ten-point Likert scale on anxiety about worries of the procedure	0=no worries, 10=I was terrified	TNE	1. Before	2 (3)	-/-
				2. During insertion	3 (3)	-

NA, not available; *STAI*, State-Trait Anxiety Inventor; *HAD*, Hospital Anxiety and Depression Scale; *IE*, impact event; *huTNE*, in clinic unsedated transnasal endoscopy; *muTNE*, mobile-based unsedated transnasal endoscopy; *EGD*, conventional upper endoscopy; *ECE*, esophageal capsule endoscopy; *TCE*, tethered capsule endomicroscopy.

Table S8. Additional patient-reported outcome data

Reference	Screening test	Instrument, Scoring Instructions and Cutoff Score for Acceptability outcome	Acceptability score: M, ± SD/SEM/ range/IQR	Acceptability score: interpretation	% preferring screening test	Willingness to undergo the procedure again afterwards (%)	Additional outcome
Blevins et al (2018)[19]	huTNE	..	..	..	..	84%	Waiting trade off method: mean days willing to wait for test result = 7.4 (SEM 1.3)
	muTNE	..	..	..	..	84%	Waiting trade off method: mean days willing to wait for test result = 7.1 (SEM 0.8)
	EGD (sedated)	..	..	..	..	100%	Waiting trade off method: mean days willing to wait for test result = 5.8 (SEM 0.7)
Chang et al (2011)[13]	TNE	..	..	..	..	89·5%	..
Eliakim et al (2004)[8]	ECE	..	..	..	73·3%	..	..
	EGD	..	..	..	6·7%	..	..
Fitzgerald et al (2020)[20]	Cytosponge-TFF3	0-10 VAS; 0=not acceptable, 10=completely acceptable	9 (IQR, 8-10) 1427 (97%) scored acceptability 5 or higher	++	..	..	..
Gupta et al (2014)[23]	TNE	..	..	..	8%	..	Baseline perception of OAC risk: 28/133 (21%)* Reasons for screening modalities to be chosen: safe/minimal risk profile (67%), low cost (50%), lack of sedation (37%) Reasons for screening modalities to be declined: lack of physician recommendation (47%), lack of current symptoms (33%), insufficient information (27%) Awareness of Barrett's oesophagus 35/136 (26%), awareness of sEGD 78/136 (57%), awareness of uTNE 24/136 (18%), awareness of ECE 23/136 (17%)
	ECE	..	..	..	56%	..	
	EGD	..	..	..	36%	..	
Kadri et al (2010)[12]	Cytosponge-TFF3	0-10 VAS; 0=worst experience, 10=best experience	7·0 (IQR, 5·0-8·0)	+	..	..	..
Mori et al (2011)[14]	TNE	..	..	..	46·0%	..	..

	UUE	..	..	..	37·9%	..	..
	EGD (sedated)	..	..	..	16·1%	..	..
Peters et al A (2020)[6]	No specific test	..	..	..	..	..	Change in predicted uptake (%):<br>Pain and discomfort:<br>Mild, -1.5%; moderate, -2.0%; Severe, -22.8%.<br>Out-of-pocket cost:<br>€25, -6.2%; €50, -10.3%; €75, -22.8%.<br>Test specificity:<br>100%, +17.1%; 90%, +12.7%; 70%, -3.2%.<br>Location:<br>At home, -0.8%; mobile unit, -1.5%; hospital, -1.0%.<br>Participants were willing to accept a 67%–70% additional risk of unnecessary follow-up testing to undergo less invasive screening.<br>Participants were willing to give up 26% test sensitivity to undergo a minimally invasive screening test.
Peters et al B (2020)[7]	EGD, TNE, cell collection device, breath analysis, blood test	..	..	..	..	..	Change in predicted uptake (%):<br>Screening test:<br>EGD, -16.1%; TNE, -12.2%; cell collection device, -6.6%; breath analysis, + 12.7%; blood test + 13.7%.<br>Test sensitivity:<br>100%, +17.6%; 90%, +10.2%; 80%, +0.4%; 70%, -15.0%, 60%, -25.0%.<br>Test specificity:<br>100%, +9.0%; 90%, +1.4%; 80%, +2.7%; 70%, -5.8%, 60%, -9.4%.<br><br>A low sensitivity would result in 25.0% decrease in expected uptake.<br>Other tests should have the following sensitivity/specificity to be equally attractive as upper endoscopy:<br>- Breath test: 80% sens, 80% spec<br>- Cell collection devices: 100% sens, 70% spec<br>- TNE 100% sens, 80% spec
Ramirez et al (2008)[11]	ECE	..	..	..	81%	..	..
	EGD	..	..	..	19%	..	..

huTNE, in clinic unsedated transnasal endoscopy; *muTNE*, mobile-based unsedated transnasal endoscopy; *EGD*, conventional upper endoscopy; *ECE*, esophageal capsule endoscopy; *TCE*, tethered capsule endomicroscopy; *UUE*, unsedated ultrathin endoscopy.
*measured as proportion of participants who believed they were at higher risk of developing oesophageal cancer in the next 10 years in comparison to individuals of similar age, gender, race.

Table S9. Summary patient-reported outcomes per screening test

Screening test	Interpretation tolerability scores in individual studies	Summary tolerability	Interpretation anxiety scores in individual studies	Summary anxiety	Interpretation acceptability scores in individual studies	Summary acceptability
Sedated conventional upper endoscopy	Blevins et al:[19] ++<br>Mori et al:[14] +	++	Blevins et al:[19] -/-	-/-	..	..
Unsedated conventional upper endoscopy	Essink et al:[10] +/-	+/-	Essink et al:[10] -	-	..	..
Unsedated ultrathin oral endoscopy	Mori et al:[14] +	+	..	..		
Unsedated transnasal endoscopy	Blevins et al:[19] +<br>Chak et al:[16] +<br>Chang et al:[13] +<br>Mori et al:[14] ++<br>Peery et al:[15] ++	+	Blevins et al:[19] -<br>Chak et al:[16] +/-<br>Chang et al:[13] -<br>Peery et al:[15] -	-	..	..
Wireless capsule endoscopy	Chak et al:[16] ++<br>Ramirez et al:[11] +	++	Chak et al:[16] -	-	..	..
Tethered microendoscopy	Gora et al:[18] +/-	+/-	Gora et al:[18] -	-	..	..
Cytosponge-TFF3	..	..	Kadri et al:[12] -	-	Fitzgerald et al:[20] ++<br>Kadri et al:[12] +	++

-/-, very low tolerability/anxiety/acceptability; –, low tolerability/anxiety/acceptability; +/-, medium tolerability/anxiety/acceptability; +, high tolerability/anxiety/acceptability; ++, very high tolerability/anxiety/acceptability.

5. References

1. Rosenstock IM, Strecher VJ, Becker MH. Social learning theory and the Health Belief Model. *Health Educ Q* 1988; **15**(2): 175-83.
2. Myers RE. Decision counseling in cancer prevention and control. *Health Psychol* 2005; **24**(4S): S71-7.
3. Tan WK, Muldrew B, Khan Z, Fitzgerald RC. A cross-sectional analysis of facebook comments to study public perception of a new diagnostic test called the Cytosponge. *Diseases of the Esophagus* 2019; **32**(1).
4. Freeman M, Offman J, Walter FM, Sasieni P, Smith SG. Acceptability of the Cytosponge procedure for detecting Barrett's oesophagus: a qualitative study. *BMJ Open* 2017; **7**(3): e013901.
5. McGoran J, Bennett A, Cooper J, et al. Acceptability to patients of screening disposable transnasal endoscopy: qualitative interview analysis. *BMJ Open* 2019; **9**(12): e030467.
6. Peters Y, van Grinsven E, van de Haterd M, van Lankveld D, Verbakel J, Siersema PD. Individuals' Preferences for Esophageal Cancer Screening: A Discrete Choice Experiment. *Value Health* 2020; **23**(8): 1087-95.
7. Peters Y, Siersema PD. Public Preferences and Predicted Uptake for Esophageal Cancer Screening Strategies: A Labeled Discrete Choice Experiment. *Clin Transl Gastroenterol* 2020; **11**(11): e00260.
8. Eliakim R, Yassin K, Shlomi I, Suissa A, Eisen GM. A novel diagnostic tool for detecting oesophageal pathology: the PillCam oesophageal video capsule. *Aliment Pharmacol Ther* 2004; **20**(10): 1083-9.
9. Wilkins T, Gillies RA. Office-based unsedated ultrathin esophagoscopy in a primary care setting. *Ann Fam Med* 2005; **3**(2): 126-30.
10. Essink-Bot ML, Kruijshaar ME, Bac DJ, et al. Different perceptions of the burden of upper GI endoscopy: an empirical study in three patient groups. *Qual Life Res* 2007; **16**(8): 1309-18.
11. Ramirez FC, Akins R, Shaukat M. Screening of Barrett's esophagus with string-capsule endoscopy: a prospective blinded study of 100 consecutive patients using histology as the criterion standard. *Gastrointest Endosc* 2008; **68**(1): 25-31.
12. Kadri SR, Lao-Sirieix P, O'Donovan M, et al. Acceptability and accuracy of a non-endoscopic screening test for Barrett's oesophagus in primary care: cohort study. *BMJ* 2010; **341**: c4372.
13. Chang JY, Talley NJ, Locke GR, 3rd, et al. Population screening for barrett esophagus: a prospective randomized pilot study. *Mayo Clin Proc* 2011; **86**(12): 1174-80.
14. Mori A, Ohashi N, Yoshida A, et al. Unsedated transnasal ultrathin esophagogastroduodenoscopy may provide better diagnostic performance in gastroesophageal reflux disease. *Dis Esophagus* 2011; **24**(2): 92-8.
15. Peery AF, Hoppo T, Garman KS, et al. Feasibility, safety, acceptability, and yield of office-based, screening transnasal esophagoscopy (with video). *Gastrointest Endosc* 2012; **75**(5): 945-53 e2.
16. Chak A, Alashkar BM, Isenberg GA, et al. Comparative acceptability of transnasal esophagoscopy and esophageal capsule esophagoscopy: a randomized, controlled trial in veterans. *Gastrointest Endosc* 2014; **80**(5): 774-82.
17. Sami SS, Dunagan KT, Johnson ML, et al. A randomized comparative effectiveness trial of novel endoscopic techniques and approaches for Barrett's esophagus screening in the community. *Am J Gastroenterol* 2015; **110**(1): 148-58.
18. Gora MJ, Simmons LH, Queneherve L, et al. Tethered capsule endomicroscopy: from bench to bedside at a primary care practice. *J Biomed Opt* 2016; **21**(10): 104001.
19. Blevins CH, Egginton JS, Shah ND, Johnson ML, Iyer PG. Comparative Assessment of Patient Preferences and Tolerability in Barrett Esophagus Screening: Results From a Randomized Trial. *J Clin Gastroenterol* 2018; **52**(10): 880-4.
20. Fitzgerald RC, di Pietro M, O'Donovan M, et al. Cytosponge-trefoil factor 3 versus usual care to identify Barrett's oesophagus in a primary care setting: a multicentre, pragmatic, randomised controlled trial. *Lancet* 2020; **396**(10247): 333-44.
21. Bridges JF, Hauber AB, Marshall D, et al. Conjoint analysis applications in health--a checklist: a report of the ISPOR Good Research Practices for Conjoint Analysis Task Force. *Value Health* 2011; **14**(4): 403-13.
22. Chad-Friedman E, Coleman S, Traeger LN, et al. Psychological distress associated with cancer screening: A systematic review. *Cancer* 2017; **123**(20): 3882-94.
23. Gupta M, Beebe TJ, Dunagan KT, et al. Screening for Barrett's esophagus: results from a population-based survey. *Dig Dis Sci* 2014; **59**(8): 1831-50.
