# Supplementary material for: Public acceptance and uptake of oesophageal adenocarcinoma screening strategies: A mixed-methods systematic review
Source: eClinicalMedicine. 2022 Apr 4;46:101367. doi: 10.1016/j.eclinm.2022.101367 (PMC8987366; doi:10.1016/j.eclinm.2022.101367)
Supplement: Supplementary file 2 [file mmc2.docx]

Appendix
